# High-efficacy serum biomarkers PCSK9 and LCAT predict cognitive impairment in Parkinson’s disease

**DOI:** 10.3389/fpsyg.2026.1789929

**Published:** 2026-04-15

**Authors:** Mingyu Su, Tianshu Yang, Xinrui Fan, Xiyu Qiang, Zihan Liao, Heng Gu, Linyun Gao, Rui Chen, Chuanxi Tang, Chunyan Mu

**Affiliations:** 1 Department of Neurobiology, Xuzhou Key Laboratory of Neurobiology, Xuzhou Medical University, Xuzhou, Jiangsu, China; 2 School of Public Health, Xuzhou Medical University, Xuzhou, Jiangsu, China; 3 First School of Clinical Medicine, Xuzhou Medical University, Xuzhou, Jiangsu, China; 4 Department of Neurology, Huaian Second People's Hospital, Huaian, Jiangsu, China; 5 Xuzhou Oriental People’s Hospital Affiliated to Xuzhou Medical University, Xuzhou, Jiangsu, China

**Keywords:** cognitive function, lipid metabolism, neuroinflammation, Parkinson’s disease, serum protein biomarkers

## Abstract

**Background:**

Cognitive impairment (CI) is a prevalent and debilitating non-motor symptom in Parkinson’s disease (PD), yet reliable early diagnostic biomarkers are lacking. This study aimed to identify serum biomarkers associated with PD-CI and investigate the synergistic contributions of lipid metabolism and inflammatory signaling.

**Methods:**

In this retrospective, cross-sectional study, six candidate proteins (INPP5D, FLNA, ICAM-1, PCSK9, JAK1, and LCAT) were selected based on our previously published discovery-phase serum proteomics analysis and were quantified via ELISA in an independent cohort of 75 PD patients and 35 age-matched healthy controls (HCs). All participants underwent comprehensive cognitive assessments (MoCA, MMSE, CDR). Multivariate regression, receiver operating characteristic (ROC) analysis, and bioinformatics tools were employed to evaluate diagnostic potential and pathway associations.

**Results:**

PD patients showed significantly lower MoCA and MMSE scores than HC, accompanied by elevated serum ICAM-1, PCSK9, and JAK1, and decreased INPP5D and FLNA. Notably, as MoCA scores declined, serum ICAM-1, PCSK9, JAK1, and LCAT levels gradually increased, while INPP5D and FLNA decreased. ROC analysis indicated that these biomarkers, particularly PCSK9 and LCAT, effectively distinguished PD-NC from PD-CI. Bioinformatics analyses highlighted focal adhesion and JAK-STAT signaling as key pathways, with ICAM1 and ITGB2 as central nodes in the protein–protein interaction network.

**Conclusion:**

All six serum biomarkers showed potential in distinguishing PD-NC from PD-CI, with PCSK9 and LCAT being the most effective. The findings propose a pathogenic cascade integrating neuroinflammation, lipid metabolism, and cell adhesion dysfunction, offering new mechanistic insights and potential avenues for early diagnosis and therapeutic intervention in PD-CI.

## 1 Introduction

Parkinson’s disease (PD) is a common neurodegenerative disorder. With the intensification of population aging, the incidence of PD continues to rise, attracting widespread attention. The clinical manifestations of PD include motor and non-motor symptoms: the motor symptoms primarily consist of resting tremor, bradykinesia, rigidity, and postural instability; the non-motor symptoms encompass sleep disorders, cognitive impairment (CI), anxiety, depression, and hyposmia (Hayes, 2019). Among these, early CI is the most common non-motor symptom, which can manifest 5–10 years before the onset of motor symptoms (Yang et al., 2016), and some patients may progress to dementia, resulting in severe impairment of daily living, occupational, and financial functioning, which is further exacerbated by comorbid depression (Leroi et al., 2012; Giannouli and Tsolaki, 2019). Notably, the pathological progression of PD typically initiates several years before the manifestation of clinical symptoms such as CI, by which time significant neuronal and synaptic damage has already occurred (Wilson et al., 2023). Therefore, utilizing biomarkers to identify affected individuals early in the pre-symptomatic stage could enable the efficient development and evaluation of disease-modifying therapies for PD.

In recent years, research on blood biomarkers has provided new directions for the early diagnosis of PD (EBio, 2021; Alcolea et al., 2023). Based on multi-omics studies, we previously screened six biomarkers with potential diagnostic value: Inositol Polyphosphate-5-Phosphatase D (INPP5D), Filamin A (FLNA), Intercellular Adhesion Molecule 1 (ICAM-1), Proprotein Convertase Subtilisin/Kexin Type 9 (PCSK9), Janus Kinase 1 (JAK1), and Lecithin-Cholesterol Acyltransferase (LCAT). These proteins are involved in key pathological mechanisms of PD, such as neuroinflammation, lipid metabolism, and signal transduction, and may constitute a molecular network underlying early CI. INPP5D, as a member of the INPP5 family, has been confirmed to be associated with an increased risk of dementia, and its inhibition may exert neuroprotective effects (Lin et al., 2023). INPP5F, another member of the same family, has also been found to be associated with PD risk (Xue et al., 2022); studies suggest that modulating INPP5F expression in brain tissue may increase the risk of dementia. These findings indicate that members of the INPP5 family may play synergistic roles in neurodegenerative diseases. FLNA, a cytoskeletal regulatory protein, has been proposed as a therapeutic target for dementia (Burns and Wang, 2017), and its gene polymorphisms may be associated with PD susceptibility (Odumpatta and Mohanapriya, 2020). Regarding inflammatory mechanisms, ICAM-1, as an important pro-inflammatory molecule, has been confirmed to exacerbate neuroinflammatory responses in PD patients (Zhang et al., 2024). Clinical studies have found that elevated levels of ICAM-1 in the cerebrospinal fluid (CSF) of PD patients are significantly correlated with clinical symptom scores and neurodegenerative markers, suggesting that ICAM-1 may be involved in the inflammatory and cognitive decline processes of PD (Lerche et al., 2023). Abnormal activation of the JAK1/STAT signaling pathway is closely related to neuroinflammation, and recent research indicates that JAK1 may become a potential therapeutic target for PD (Khodir et al., 2024). Lipid metabolism dysregulation is another important feature of neurodegenerative diseases. PCSK9, as a key regulator of lipid metabolism, shows significantly elevated concentrations in the CSF of dementia patients (Zimetti et al., 2017). LCAT, as a key enzyme in lipid metabolism, has been confirmed to participate in the cognitive decline process in dementia patients (Hong et al., 2022).

However, there is currently a lack of systematic research on the diagnostic application value of the aforementioned six serum biomarkers, including INPP5D, ICAM-1, and LCAT, for early CI in PD, and their combined diagnostic efficacy and clinical feasibility remain to be elucidated. This study, employing a cross-sectional design, deeply revealed the differences in serum levels of INPP5D, FLNA, ICAM-1, PCSK9, JAK1, and LCAT in PD onset and among PD patients with varying degrees of CI, and for the first time evaluated the clinical application potential of these biomarkers for early PD diagnosis and PD cognitive screening. Furthermore, we preliminarily constructed a synergistic interaction network among these several substances, revealing potential synergistic effects among lipid metabolism, inflammation, and genetic factors, thereby providing an important theoretical basis for the early diagnosis and precision treatment of PD.

## 2 Methods

This experiment was a retrospective, case–control study. The study was conducted in accordance with the Declaration of Helsinki (as revised in 2024). The study was approved by the ethics committee of the Affiliated Hospital of Xuzhou Medical University (XYFY2020-KL023-01). All participants signed the informed consent form voluntarily to participate in this study.

### 2.1 Participants

We enrolled 75 participants with PD and 35 participants as healthy Control (HC).

#### 2.1.1 Inclusion and exclusion criteria

*PD Patients were included if they met the following criteria*: (I) Clinical diagnosis confirmed by the UK Parkinson’s Disease Society Brain Bank Criteria; (II) Disease severity at Hoehn and Yahr (H–Y) stage <3; (III) Availability of serum material for analysis; (IV) Voluntary participation with provision of signed informed consent; (V) Ability to be evaluated through relevant standardized assessment scales; (VI) Receiving standardized anti-PD drug treatment under the guidance of a neurology specialist. *Participants were excluded if they had any history of*: (I) Diagnosis of secondary or atypical Parkinsonism. This was objectively confirmed through: (a) brain magnetic resonance imaging to exclude prominent cortical/subcortical infarcts, cerebral small vessel disease, or atypical signs (e.g., midbrain, cortical, or cerebellar atrophy); and (b) 123I-FP-CIT SPECT imaging to confirm nigrostriatal dopaminergic degeneration; (II) Significant decline in vision or hearing that would considerably impact the reliability of scale test results; (III) Presence of severe systemic/physical conditions potentially affecting serum biomarker levels, including kidney, liver, rheumatologic, and neoplastic diseases, or a history of drug or alcohol abuse; (IV) Presence of obvious depression or agitation (HAMA ≥24, HAMD ≥34); (V) Diagnosis of dementia according to current criteria; (VI) Presence of altered laboratory parameters, including serum creatinine levels (>1.2 mg/dL) or elevated liver function parameters (aspartate transaminase or alanine transaminase >50 U/L).

*HC people were included if they met the following criteria*: (I) Age approximately matched to the PD group participants (±5 years); (II) No history of Parkinson’s disease, Alzheimer’s disease, stroke, epilepsy, multiple sclerosis, brain tumors, or any post-traumatic brain disorders known to affect the nervous system; (III) No family history of Parkinson’s disease; (IV) No history of long-term drug use: specifically, did not take any medications that might affect neurological function or the study’s target biomarkers for a prolonged period; (V) Normal cognitive function; (VI) Voluntary participation with provision of signed informed consent. *Participants were excluded if they had any history of*: To ensure the absence of potential confounding factors, the HC cohort also met the PD group’s exclusion criteria no. (III) and (VI), pertaining to severe systemic diseases and abnormal laboratory parameters. All PD patients underwent a standardized neurological examination, including motor assessment using the Movement Disorders Society Unified Parkinson’s Disease Rating Scale part III (MDS-UPDRS-III), global cognition assessment (MMSE), and Mini Nutritional Assessment. The levodopa equivalent daily dose (LEDD) was calculated at baseline according to established conversion factors.

#### 2.1.2 Clinical evaluation (behavioral and cognitive assessments)

We collected the general demographic data and clinical characteristics of the participants, including age, gender, educational level, alcohol and tobacco usage, etc. The severity of movement disorders was evaluated using the H-Y staging system. To avoid the influence of symptom fluctuations on the assessment results, all patients were underwent cognitive assessment during the stable period, including The Mini-Mental State Examination (MMSE) and The Montreal Cognitive Assessment (MoCA) as global screening instruments, both of which are widely validated for detecting cognitive impairment in Parkinson’s disease populations. MoCA is particularly sensitive for identifying early executive and visuospatial deficits, which are common in PD-related cognitive dysfunction. and the Clinical Dementia Rating (CDR) was additionally employed to characterize dementia severity and functional impairment, allowing for clinical staging of cognitive decline. The diagnosis of cognitive status in patients with Parkinson’s disease was determined based on consensus reached through neuropsychological tests (MMSE, MoCA, CDR), neurological examinations conducted by doctors, and the assessment of the patient’s daily living conditions.

#### 2.1.3 Grouping based on cognition function level

We initially identified the subjects with MMSE scores above 26 as those without CI. Subsequently, by combining the MoCA assessment and the clinicians’ evaluations of the patients’ daily cognition, we determined whether PD patients had CI. Then, we began to analyze the cognitive characteristics of different groups and reveal the comprehensive serum metabolism and proteomics features of these two groups. Based on the clinicians’ judgments, we grouped the subjects, and then the researchers conducted data analysis to avoid selection bias in our study. We found that a group of subjects with relatively good cognitive performance and little impact on their lives were defined as the Parkinson’s disease cognitive healthy group (PD-N). On the contrary, the subjects with MMSE scores below 26 and poor performance in MoCA and daily life were defined as the Parkinson’s disease cognitive impairment group (PD-CI).

#### 2.1.4 Serum sample preparation

On the morning of the patient’s enrollment (7.00 a.m.—10.00 a.m.), peripheral venous blood was collected by vacuum blood collection tubes in a fasting state. The supernatant was centrifuged at 3000 revolutions per minute for 15 min at 4 °C, then collected and stored at −80 °C until analysis.

#### 2.1.5 ELISA

The candidate biomarkers were selected based on our previously published discovery-phase serum proteomics study on cognitive dysfunction in Parkinson’s disease. These proteins were prioritized due to both their statistical significance and their potential mechanistic relevance to neurodegenerative processes. Specifically, lipid metabolism (LCAT, PCSK9), neuroimmune signaling (INPP5D, JAK1, ICAM1), and cytoskeletal or synaptic structural regulation (FLNA) have all been implicated in pathways associated with cognitive decline and neurodegeneration. Serum concentrations of six candidate biomarkers—INPP5D, FLNA, ICAM-1, PCSK9, JAK1, and LCAT—were quantified using commercially available enzyme-linked immunosorbent assay (ELISA) kits according to the protocols (MEIMIAN Biotechnology Co., Ltd., China). Diluted serum samples and standards were added to antibody-precoated wells and incubated. After washing, enzyme-conjugated detection antibodies were applied, followed by substrate addition for colorimetric development. Absorbance was measured using a microplate reader, and protein concentrations were determined via standard curves. All assays included internal quality controls to ensure reproducibility and analytical accuracy.

### 2.2 Combined proteomics and metabolomics analysis

#### 2.2.1 KEGG pathway analysis of sphingolipid and arachidonic acid metabolism

The target gene list was compiled and analyzed using the DAVID bioinformatics resource.^1^ These genes were imported into DAVID, and KEGG pathway analysis was selected as the functional annotation category. The system calculated the Gene Ratio (representing the proportion of input genes mapped to each pathway) to evaluate enrichment significance. For every significantly enriched pathway, the number of associated genes (Count) and corresponding *p*-value were recorded. The −log~10 ~ (*p*-value) transformation was applied to normalize statistical significance. Finally, bubble plots were generated using ggplot2 in R or comparable visualization tools, where bubble size corresponds to Count and color intensity reflects the statistical significance.

#### 2.2.2 Protein interaction

Serum differential protein expression data from the Parkinson’s disease (PD) group were imported into the STRING online database.^2^ A protein–protein interaction (PPI) network was constructed using an interaction confidence score threshold of 0.4. The resulting network data were exported and imported into Cytoscape software for visualization and topological analysis. Hub genes were subsequently identified and filtered based on their degree centrality values.

### 2.3 Statistical analysis

We used independent sample *t*-tests to compare the mean differences of various biomarkers and clinical indicators between the PD group and the HC group. Before the analysis, normality tests were conducted on the data. Parametric tests were applied to data that conformed to a normal distribution, while non-parametric tests were used for data that did not, to ensure the scientific and accurate nature of the analysis results and effectively identify significant differences between the two groups. Analysis of variance (ANOVA) was employed to deeply evaluate the variations among different CI subgroups of PD (PD-Normal, PD-Mild, PD-Moderate, PD-Severe). Similarly, normality tests were conducted before the analysis. Parametric ANOVA was used for groups that conformed to a normal distribution, while non-parametric tests were applied to those that did not, to reveal significant differences among the subgroups and assist in understanding the clinical heterogeneity of PD. Given the possibility of non-normal distribution of data in the study, the Spearman correlation analysis method was adopted to assess the correlation between the degree of CI and the MoCA score, effectively revealing the potential association between the two variables. Receiver operating characteristic (ROC) curve analysis was used to evaluate the associative ability of serum protein content for PD and CI in PD patients. By calculating the area under the curve (AUC), the optimal cut-off value, sensitivity, and specificity, the efficacy of serum protein as a diagnostic biomarker was determined, providing a reference basis for clinical practice.

## 3 Results

### 3.1 Demographic characteristics and basic clinical information

A total of 35 HC people and 75 PD patients were enrolled. Demographic and clinical characteristics are presented in Tables 1–4. There were no significant differences between the two groups in terms of gender, smoking history, alcohol consumption history, hypertension history, stroke history, or diabetes history (chi-square test, *p* > 0.3) (Table 1). However, cognitive assessments demonstrated significant differences: the PD group exhibited significantly lower MoCA and MMSE scores compared to the HC group, along with higher Hamilton Anxiety Rating Scale (HAMA) and Hamilton Depression Rating Scale (HAMD) scores, indicating greater severity of anxiety and depression in the PD group (Table 1). Gender-specific analysis revealed that males had a higher proportion of individuals with normal MMSE scores and a higher mean MMSE score than females (Table 2). Regarding MoCA scores, males also showed a slightly higher proportion with mild CI and a marginally higher mean score (Table 3). Conversely, CDR scores indicated a lower mean score in males compared to females, with a certain proportion of females exhibiting severe CI (Table 4). Advancing age was associated with declines in MMSE, MoCA, and CDR scores (Tables 2–4). Higher educational attainment was associated with relatively higher scores across all three assessments. Notably, the PD group was significantly older than the HC group (67 vs. 61 years, *p* < 0.0001) (Table 1). This age difference was considered in subsequent interpretations.

**Table 1:** Demographic characteristics and basic clinical information.

Variables	PD whole (*n* = 75)	HC whole (*n* = 35)	*p* value
Age (years)	67.29 ± 8.416	61.17 ± 6.542	<0.0001****
Sex (male)	57.33%	42.86%	0.5892
Disease duration (years)	5.021 ± 4.601	–	<0.0001****
HY stage	1.992 ± 0.8685	–	<0.0001****
MoCA	19.68 ± 6.367	26.23 ± 2.390	<0.0001****
HAMA	5.899 ± 5.317	1.778 ± 1.899	<0.0001****
HAMD	7.662 ± 5.573	1.222 ± 1.758	<0.0001****
Smoking history (have)	16.00%	13.89%	>0.9999
Alcohol consumption history (have)	22.67%	13.89%	0.9920
Geriatric depression scale	6.986 ± 4.704	–	<0.0001****
MMSE	24.95 ± 5.141	28.83 ± 1.043	<0.0001****
History of hypertension (have)	22.67%	41.67%	0.3436
History of diabetes mellitus (have)	8.00%	19.44%	0.9076
History of stroke (have)	10.67%	2.78%	0.9981
Cdr			

n_PD_ = 75, n_HC_ = 35. ^****^p < 0.001. PD, Parkinson’s disease; PD-CI, PD with cognitive impairment; PD-N, PD without cognitive impairment; MoCA, Montreal Cognitive Assessment; MMSE, Mini-Mental State Examination; HAMA, Hamilton Anxiety Scale; HAMD, Hamilton Depression Scale.

**Table 2:** Analysis of factors affecting MMSE scores in PD patients.

Variables	Moderate (10–20)	Mild (21–27)	Normal (27–30)	Average
Sex				
Male	4	12	22	25.50
Female	7	6	12	24.12
Age				
44–60	–	4	3	26.29
61–75	8	10	20	24.21
76–90	2	3	6	26.09
Course of disease				
0–5	7	13	24	25.00
6–20	3	5	8	25.13
21–40	1	–	1	20.5
Degree of education				
Illiterate	6	4	7	22.82
Primary	4	5	9	24.39
Junior	–	2	7	28.22
Senior	1	6	7	25.36
University	–	1	3	26.50
H-Y installment				
1–1.5	1	3	15	27.05
2–2.5	2	8	11	26.00
3–5	2	2	4	24.88

**Table 3:** Analysis of factors affecting MoCA scores in PD patients.

Variables	Severe (0–9)	Mild (10–20)	Moderate (21–25)	Normal (26–30)	Average
Sex					
Male	1	20	9	8	20.11
Female	6	7	5	8	19.04
Age					
44–60	–	2	2	3	22.00
61–75	5	20	8	5	19.31
76–90	1	5	4	1	19.73
Course of disease					
0–5	3	21	8	12	19.98
6–20	2	6	4	4	19.38
21–40	1	–	1	–	15.00
Degree of education					
Illiterate	5	4	3	3	16.23
Primary	–	13	2	3	18.44
Junior	–	3	–	6	23.89
Senior	1	4	7	2	21.50
University	–	1	2	1	21.75
H-Y installment					
1–1.5	–	8	3	8	22.58
2–2.5	2	10	7	2	18.19
3–5	2	1	2	3	20.00

**Table 4:** Analysis of factors affecting CDR scores in PD patients.

Variables	Normal (0)	Mild (0–1)	Moderate (1.5)	Severe (2)	Average
Sex					
Male	24	15	1	–	0.29
Female	15	11	2	2	0.50
Age					
44–60	6	5	–	–	0.27
61–75	27	19	3	1	0.40
76–90	6	4	–	1	0.45
Course of disease					
0–5	30	19	–	–	0.28
6–20	8	5	3	2	0.67
21–40	1	1	–	–	0.25
Degree of education					
Illiterate	8	6	2	1	0.59
Primary	9	10	1	–	0.45
Junior	9	2	–	–	0.09
Senior	8	6	–	1	0.37
University	4	2	–	–	0.25
H-Y installment					
1–1.5	13	7	–	–	0.21
2–2.5	15	9	1	–	0.34
3–5	7	2	1	2	0.58

### 3.2 Comparison of serum protein contents between the PD and the HC

**Figure 1:**
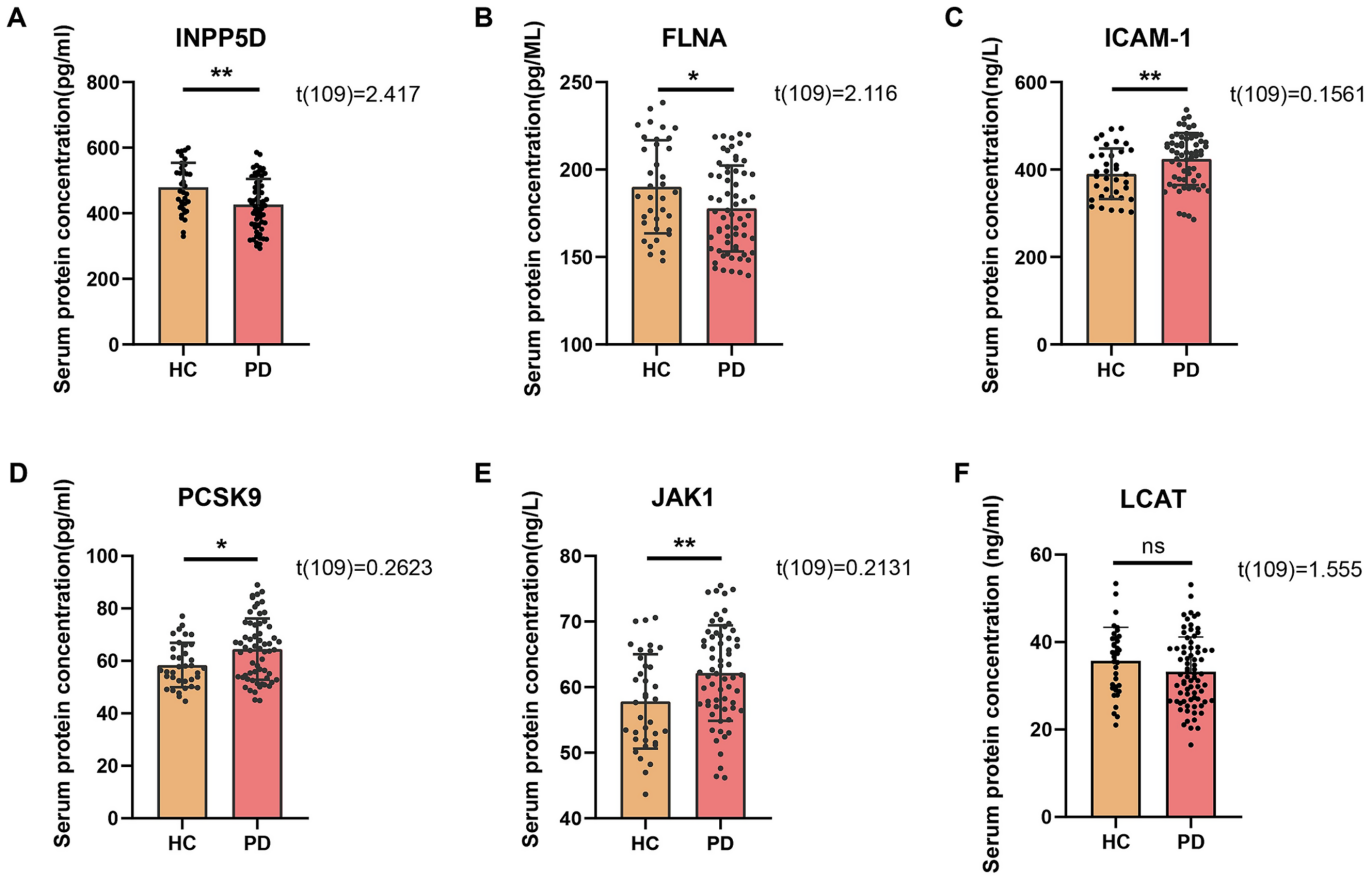
The differences in serum protein content between the PD group and the HC group, (n_PD_ = 75, n_HC_ = 35). **(A)** Comparison of serum INPP5D content between the PD group and the HC group. **(B)** Comparison of serum FLNA content between the PD group and the HC group. **(C)** Comparison of serum ICAM-1 content between the PD group and the HC group. **(D)** Comparison of serum PCSK9 content between the PD group and the HC group. **(E)** Comparison of serum JAK1 content between the PD group and the HC group. **(F)** Comparison of serum LCAT content between the PD group and the HC group. ^*^p < 0.05; ^**^p < 0.01; ns, indicates no significant difference.

Serum samples from the PD and HC groups were comparatively analyzed for differentially expressed proteins using ELISA coupled with ELISA, with a focus on the expression levels of six key proteins in Figure 1: INPP5D, FLNA, ICAM-1, PCSK9, JAK1 and LCAT. Student’s *t*-test results demonstrated that serum levels of ICAM-1, PCSK9, and JAK1 were significantly elevated in the PD group compared to the HC group (*p* < 0.01, *p* < 0.05, *p* < 0.01, respectively) (Figures 1C–E). Conversely, levels of INPP5D, FLNA were significantly reduced in the PD group (*p* < 0.01, *p* < 0.05, respectively) (Figures 1A,B). However, the level of LCAT shows no significant difference between the HC group and the PD group (Figure 1F). These findings suggest that these proteins may play important roles in the pathogenesis of Parkinson’s disease.

### 3.3 The correlation between serum protein expression levels and cognitive dysfunction in PD

**Figure 2:**
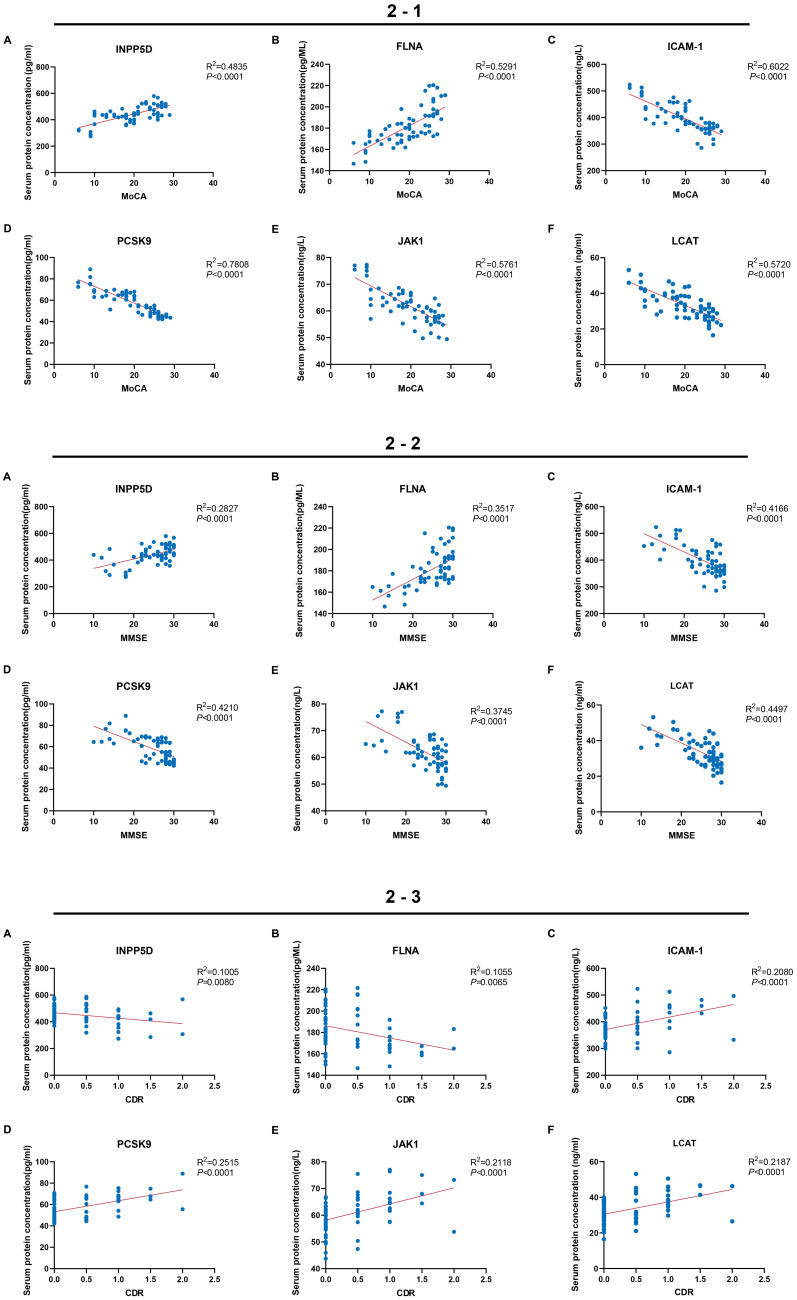
Serum protein expression levels in the PD group and MoCA, MMSE, CDR scores **(2-1)**. The correlations between the contents of serum proteins and the MoCA score (n_PD_ = 75). **(A)** INPP5D, (*R*^2^ = 0.4835, *p* < 0.001); **(B)** FLNA, (*R*^2^ = 0.5291, *p* < 0.001); **(C)** ICAM-1, (*R*^2^ = 0.6022, *p* < 0.001); **(D)** PCSK9, (*R*^2^ = 0.7808, *p* < 0.001); **(E)** JAK1, (*R*^2^ = 0.5761, *p* < 0.001); and **(F)** LCAT, (*R*^2^ = 0.5720, *p* < 0.001). **(2-2)** The correlations between the contents of serum proteins and the MMSE score, (n_PD_ = 75) **(A)** INPP5D (*R*^2^ = 0.2827, *p* < 0.001); **(B)** FLNA (*R*^2^ = 0.3517, *p* < 0.001); **(C)** ICAM-1 (*R*^2^ = 0.4166, *p* < 0.001); **(D)** PCSK9 (*R*^2^ = 0.4210, *p* < 0.001); **(E)** JAK1 (*R*^2^ = 0.3745, *p* < 0.001); and **(F)** LCAT (*R*^2^ = 0.4497, *p* < 0.001). **(2-3)** The correlations between the contents of serum proteins and the CDR score, (n_PD_ = 75). **(A)** INPP5D (*R*^2^ = 0.1005, *p* = 0080); **(B)** FLNA, (*R*^2^ = 0.1055, *p* = 0065); **(C)** ICAM-1, (*R*^2^ = 0.2080, *p* < 0.001); **(D)** PCSK9 (*R*^2^ = 0.2515, *p* < 0.001); **(E)** JAK1 (*R*^2^ = 0.2118, *p* < 0.001); and **(F)** LCAT (*R*^2^ = 0.2187, *p* < 0.001).

**Figure 3:**
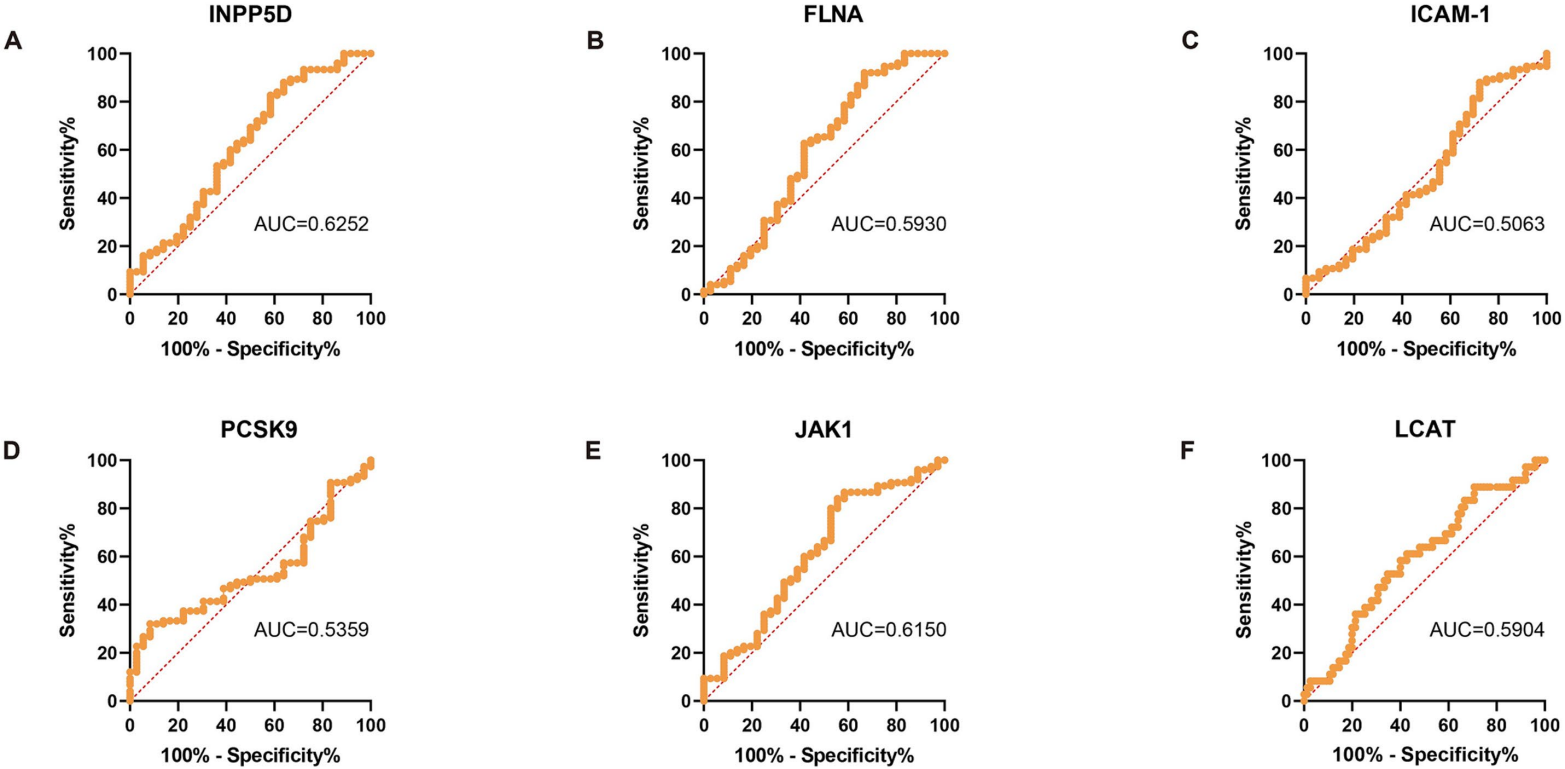
ROC analysis is used to identify the key indicators for determining whether the subjects have PD. The diagnostic accuracy of serum detection levels in determining whether the subjects have PD was evaluated using the ROC curve, (n_PD_ = 75) **(A)** INPP5D, (AUC = 0.6252); **(B)** FLNA, (AUC = 0.5930); **(C)** ICAM-1, (AUC = 0.5063); **(D)** PCSK9, (AUC = 0.5359); **(E)** JAK1, (AUC = 0.6150); and **(F)** LCAT, (AUC = 0.7553).

Analysis of the correlation between MoCA, MMSE, and CDR scores and serum protein expression revealed that the association of MoCA scores with the biomarkers was significantly stronger than that of MMSE (average increase in *R*^2^ of 42.3%) and CDR (average increase in *R*^2^ of 67.5%) (Figure 2). Specifically, INPP5D and FLNA levels showed significant positive correlations with all three cognitive scores, with the strongest correlations observed for MoCA (INPP5D: *R*^2^ = 0.4835, *p* < 0.0001; FLNA: *R*^2^ = 0.5291, *p* < 0.001) (Figures 2-1A,B). Their correlations progressively weakened with MMSE (INPP5D: *R*^2^ = 0.2827, *p* < 0.0001; FLNA: *R*^2^ = 0.3517, *p* < 0.0001) and CDR (INPP5D: *R*^2^ = 0.1005, *p* = 0.0080; FLNA: *R*^2^ = 0.1055, *p* = 0.0065) (Figures 2-2, 2-3A,B). Conversely, ICAM-1, PCSK9, JAK1, and LCAT all exhibited significant negative correlations with the cognitive scores (Figures 2-1, 2-2, 2-3C–F). Among these, LCAT displayed the strongest negative correlation with MoCA (*R*^2^ = 0.8508, *p* < 0.0001) (Figure 2-1F). The *R*^2^ values for all negatively correlated biomarkers with MoCA were significantly higher than those with the other scales (e.g., PCSK9 with MoCA showed an 85.2% higher *R*^2^ compared to MMSE) (Figure 2-1). ICAM-1 (*R*^2^ = 0.6022), PCSK9 (*R*^2^ = 0.7808), and JAK1 (*R*^2^ = 0.5761) all demonstrated strong negative correlations with MoCA (all *p* < 0.0001) (Figures 2-1C–E). Notably, the MoCA demonstrated significantly superior overall explanatory power for the six biomarkers (average *R*^2^ = 0.6370) compared to the MMSE (average *R*^2^ = 0.3531) and CDR (average *R*^2^ = 0.1806), confirming its sensitivity and reliability as a cognitive assessment tool in PD (Figure 2).

### 3.4 Compare the predictive ability of serum protein expression levels in the population for the presence or absence of PD

Building upon our prior findings of differential serum protein expression profiles between PD and HC groups and the strong correlations between MoCA scores and the six proteins (INPP5D, FLNA, ICAM-1, PCSK9, JAK1, LCAT), this study further established a diagnostic efficacy evaluation framework (Figure 3). Receiver operating characteristic (ROC) curve analysis revealed that while INPP5D (AUC = 0.6252, *p* = 0.044) and LCAT (AUC = 0.7553, *p* = 0.029) achieved statistical significance, their diagnostic performance was only marginal (Figures 3A,F). At their optimal cut-off values [INPP5D: 381.1 (sensitivity 16%, specificity 92%); LCAT: 65.17 (sensitivity 31%, specificity 97%)], both demonstrated limited clinical utility (Figures 3A,F). FLNA (AUC = 0.5930, *p* = 0.114) and JAK1 (AUC = 0.6150, *p* = 0.051) exhibited modest discriminatory ability but failed to reach statistical significance: at a cut-off of 211.2, FLNA showed high sensitivity (92%) but low specificity (33%), whereas JAK1, at a threshold of 54.74, demonstrated relatively balanced sensitivity (84%) and specificity (44%) (Figures 3B,E). Notably, ICAM-1 (AUC = 0.5063, *p* = 0.915) and PCSK9 (AUC = 0.5359, *p* = 0.541) yielded AUC values approaching random chance levels (Figures 3C,D). Their optimal cut-off values [ICAM-1: 339.7 (sensitivity 88%, specificity <30%); PCSK9: 69.97 (sensitivity 91%, specificity <30%)] suggest a potential risk of overfitting (Figures 3C,D).

### 3.5 Stepwise changes in serum levels across PD cognitive impairment stages

**Figure 4:**
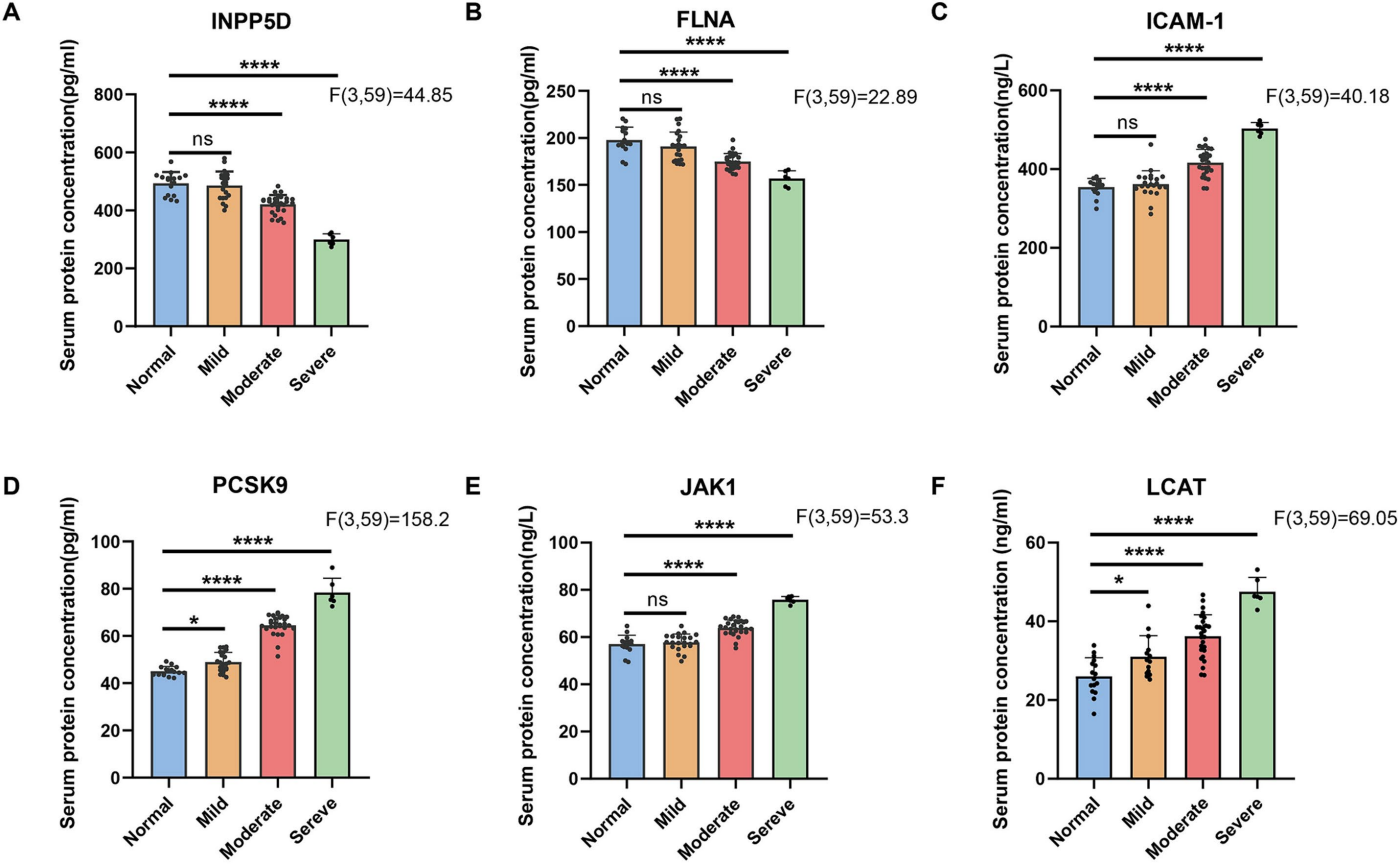
There are significant differences in serum protein content among different subgroups of PD with varying degrees of cognitive dysfunction. Comparison of serum contents among the PD-Normal group, PD-Mild group, PD-Moderate group, and PD-Severe group **(A)** INPP5D; **(B)** FLNA; **(C)** ICAM-1; **(D)** PCSK9; **(E)** JAK1; and **(F)** LCAT. ^*^p < 0.05; ^**^p < 0.01; ^***^p < 0.001; ns, indicates no significant difference.

**Figure 5:**
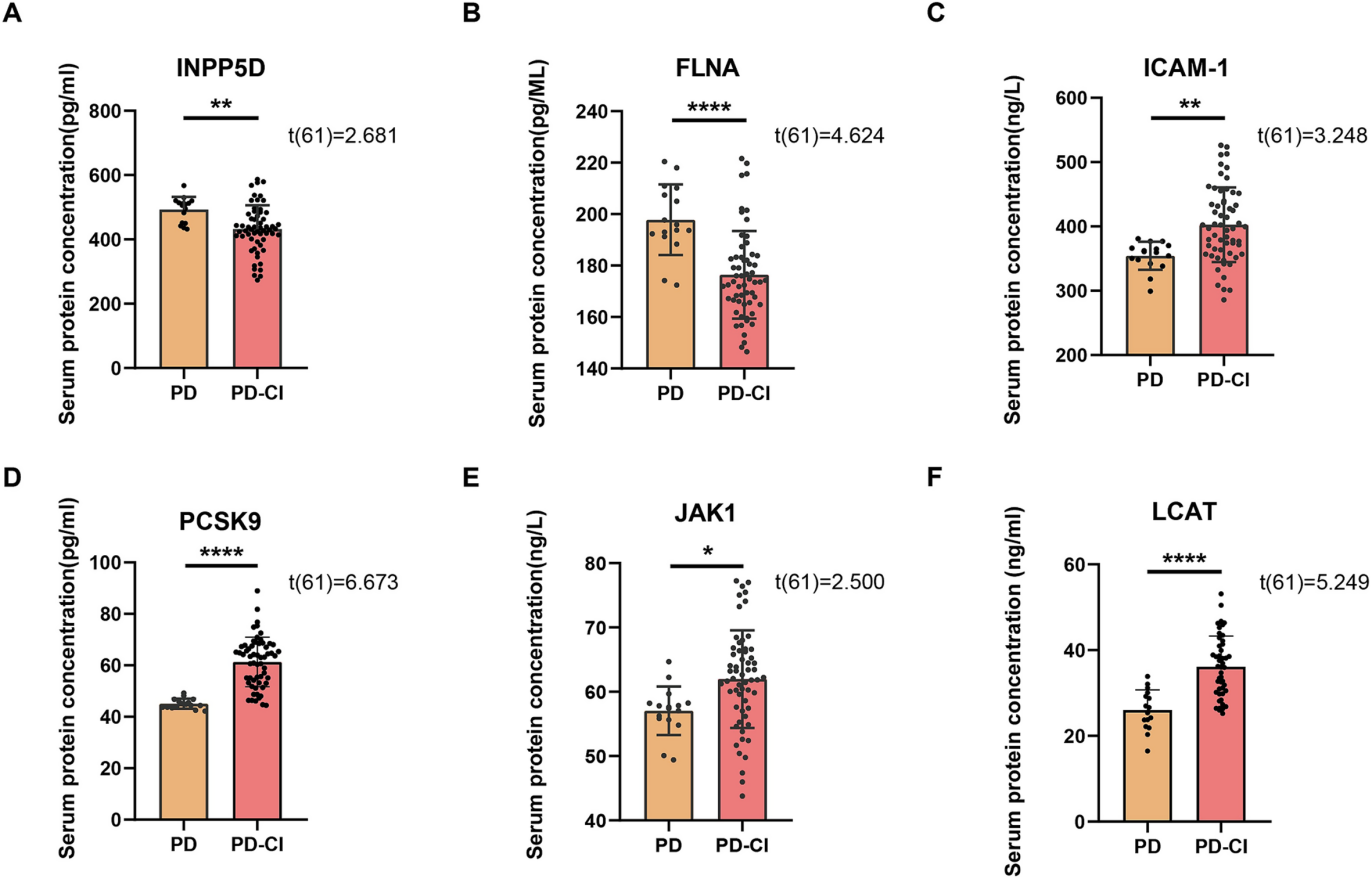
Differences in serum protein content between the PD group and the PD-CI group. Comparison of serum contents between the PD group and the PD-CI group **(A)** INPP5D; **(B)** FLNA; **(C)** ICAM-1; **(D)** PCSK9; **(E)** JAK1; and **(F)** LCAT. ^*^p < 0.05; ^**^p < 0.01; ^***^p < 0.001; ^****^p < 0.0001.

Based on MoCA scores [≥26: cognitively normal (PD-NC); <26 and ≥21: mild CI (PD-Mild); <21 and ≥10: moderate CI (PD-Moderate); <10: severe CI (PD-Severe)], PD patients were stratified into four groups (Figure 4). One-way ANOVA revealed that serum levels of INPP5D and FLNA exhibited a significant progressive decrease with worsening CI. Levels in the PD-Mild group were significantly higher than those in both the PD-Moderate and PD-Severe groups (all *p* < 0.001). Furthermore, levels in the PD-Moderate group were significantly higher than those in the PD-Severe group (INPP5D: *p* < 0.001; FLNA: *p* < 0.0001) (Figures 4A,B). Conversely, serum levels of ICAM-1, PCSK9, JAK1, and LCAT showed significant increases. Specifically, ICAM-1 levels were significantly lower in the PD-Mild group compared to the moderate and severe groups (*p* < 0.0001), and significantly lower in the PD-Moderate group compared to the PD-Severe group (*p* < 0.0001). Levels of PCSK9, JAK1, and LCAT demonstrated progressive and significant stepwise increases among the mild, moderate, and severe PD-CI subgroups (all *p* < 0.0001) (Figures 4C–F). Further independent samples *t*-tests indicated that compared to the PD-NC group, the combined PD-CI group exhibited significantly elevated serum levels of ICAM-1, PCSK9, JAK1, and LCAT (*p* < 0.01, *p* < 0.0001, *p* < 0.05, *p* < 0.0001, respectively) (Figures 5C–F), while levels of INPP5D and FLNA were significantly reduced (*p* < 0.01, *p* < 0.0001, respectively) (Figures 5A,B).

### 3.6 Compare the predictive ability of serum protein expression levels in PD patients for the presence of cognitive dysfunction

**Figure 6:**
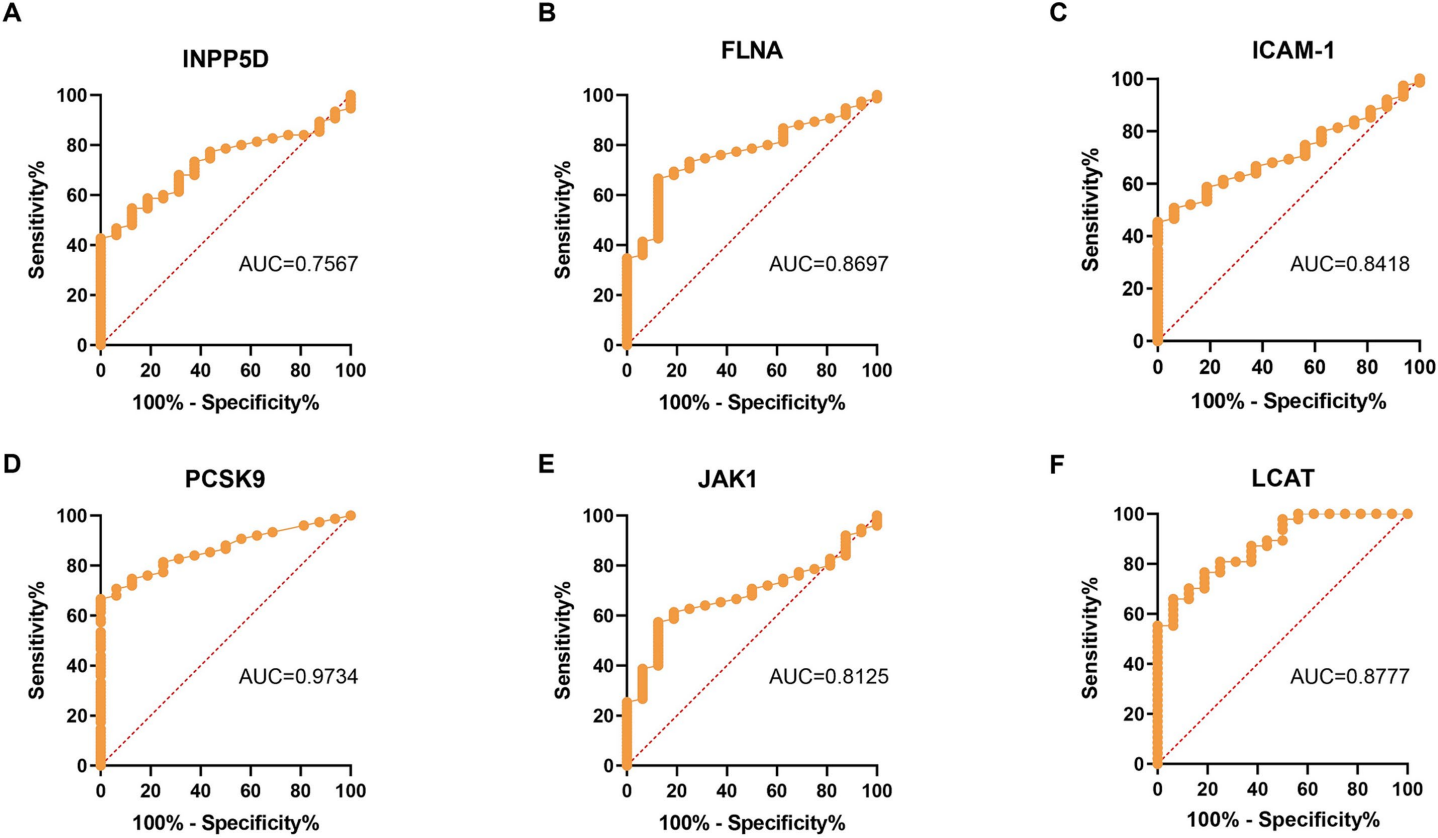
ROC analysis for identifying the key indicators for determining whether patients with PD have cognitive dysfunction. The diagnostic accuracy of serum detection levels in determining whether PD patients have cognitive dysfunction was evaluated using the ROC curve. **(A)** INPP5D (AUC = 0.7567); **(B)** FLNA (AUC = 0.8697); **(C)** ICAM-1 (AUC = 0.8418); **(D)** PCSK9 (AUC = 0.9734); **(E)** JAK1 (AUC = 0.8125); and **(F)** LCAT (AUC = 0.9428).

ROC curve analysis further elucidated the diagnostic potential of the key proteins (Figure 6). Notably, PCSK9 (AUC = 0.9734, *p* < 0.0001) and LCAT (AUC = 0.9428, *p* = 0.0002) demonstrated outstanding predictive power. At their optimal cut-off values [PCSK9: 48.48 (sensitivity 71%, specificity 94%); LCAT: 48.47 (sensitivity 74%, specificity 94%)], both biomarkers combined high specificity with clinically acceptable sensitivity (Figures 6D,F). FLNA (AUC = 0.8697, *p* = 0.0008) also achieved a favorable balance of sensitivity (68%) and specificity (81%) at a cut-off of 188.6 (Figure 6B). While INPP5D (AUC = 0.7567, *p* = 0.005) and JAK1 (AUC = 0.8125, *p* = 0.021) reached statistical significance, their sensitivities were relatively lower (44 and 63%, respectively) (Figures 6A,E). Importantly, ICAM-1 exhibited predictive performance approaching random chance levels (AUC = 0.5063, *p* = 0.915), with its lack of statistical significance further indicating a weak association with PD-CI (Figure 6C).

### 3.7 Integration of pathways and network topology analysis reveals the cooperative regulatory network and therapeutic targets in the mechanism of PD

**Figure 7:**
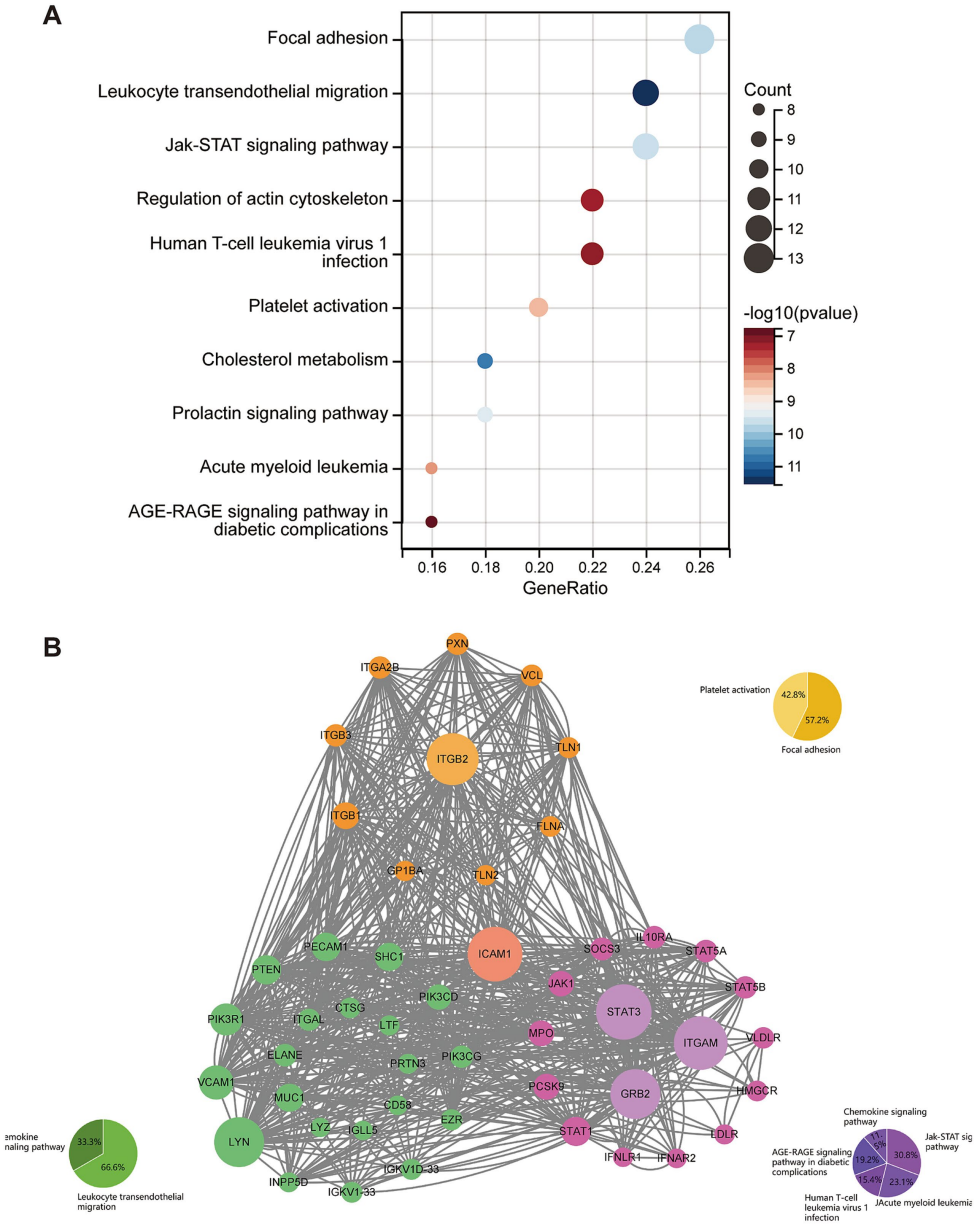
Analysis of the gene proportions of each biological pathway and the interactions of related genes. **(A)** The proportion of genes in each biological pathway and their statistical significance. The *x*-axis represents the gene ratio (GeneRatio), and the *y*-axis lists the significantly enriched pathways related to PD. The color of the points [−log_10_ (*p* value)] reflects the significance of pathway enrichment (the darker the color, the more significant); the size of the points corresponds to the gene count (Count) in the pathway (the larger the point, the more genes involved). **(B)** Analysis of the interaction between cell signaling pathway networks and related genes. The nodes represent the differential proteins, and the colors indicate the corresponding pathways. The size of the nodes reflects the importance within the network.

Pathway enrichment analysis results were visualized using a bubble plot (*x*-axis: GeneRatio; *y*-axis: Pathway name; Bubble color: −log₁₀(*p*-value); Bubble size: Count). The results revealed significant enrichment (*p* < 0.001) for the “Focal adhesion” (GeneRatio = 0.26) and “Leukocyte transendothelial migration” (GeneRatio = 0.25) pathways. “Focal adhesion” exhibited the largest bubble size (Count = 13), indicating the broadest involvement of its associated genes. Notably, while the “JAK-STAT signaling pathway” showed a moderate GeneRatio (0.18), it displayed the darkest color (*p* = 1.2 × 10^−5^), signifying the strongest statistical significance (Figure 7A). Subsequent network topology analysis identified core hub nodes ICAM1 and ITGB2. These hubs connected multiple pathways, including JAK–STAT and chemokine signaling, via multiple edges, forming a cross-pathway regulatory network. This feature aligns with the enrichment analysis: although the “Platelet activation” pathway constituted 42.8% of the module genes, it formed a functional module with the “Focal adhesion” pathway (constituting 57.2%) through shared key genes such as STAT3 and JAK1. This reveals a synergistic regulatory mechanism between platelet activation and cell adhesion in the pathological process. Network density analysis confirmed that the average connectivity of the central nodes (6.8) was significantly higher than the overall network average (3.2) (*p* = 0.007), highlighting the critical structural role of the ICAM1-ITGB2 axis in maintaining network stability (Figure 7B).

## 4 Discussion

Cognitive impairment (CI) is a common non-motor symptom in patients with Parkinson’s disease (PD). Epidemiological studies indicate that approximately 30% of PD patients exhibit varying degrees of CI at the time of diagnosis, and as the disease progresses, about 75% will eventually develop dementia (Aarsland and Kurz, 2010). Cognitive decline not only accelerates the worsening of motor symptoms but also leads to significant deterioration in patients’ daily living abilities (Lawson et al., 2016); related research confirms that PD with cognitive impairment (PD-CI) increases the 5-year mortality rate by 3–5-fold (Levy et al., 2002). Neuropathological studies reveal that by the time cognitive symptoms appear, irreversible synaptic damage and neuronal loss have already occurred in the patient’s brain (Wilson et al., 2023). Therefore, developing reliable biomarkers capable of identifying individuals at high risk for PD-CI before clinical symptoms manifest holds significant clinical value. This study employed a multidimensional research approach, conducting systematic analyses from demographic characteristics and clinical indicators to serum proteomics. The results elucidate the important value of six serum protein biomarkers—INPP5D, FLNA, ICAM-1, PCSK9, JAK1, and LCAT—in PD diagnosis and cognitive function assessment, and propose for the first time the “neuroinflammation-lipid metabolism-cell adhesion” cascade pathological hypothesis, providing a new theoretical basis and therapeutic targets for the early diagnosis and targeted intervention of PD.

Regarding baseline characteristics analysis, this study rigorously matched demographic characteristics and clinical histories between the PD group and the HC group. Statistical results showed no significant differences between the two groups in terms of gender, smoking history, alcohol consumption history, hypertension history, stroke history, or diabetes history, ensuring the comparability of the study subjects. Notably, PD patients had significantly lower MoCA and MMSE scores in cognitive function assessments and higher HAMA and HAMD scores in anxiety and depression assessments compared to the HC group. This is consistent with previous research (Zeng et al., 2023), further confirming that PD patients not only present with typical motor symptoms but also experience significant CI and emotional disorders, which collectively contribute to a substantial decline in their quality of life.

Through multifactorial regression analysis, we systematically evaluated the influencing factors of cognitive function in PD patients. The results revealed that female PD patients had a significantly higher degree of CI than males; this gender difference may be related to the weakening of neuroprotective effects caused by declining estrogen levels after menopause (Schaffner et al., 2025). Furthermore, increasing age and longer disease duration aggravated CI, while higher education levels exerted a certain protective effect on cognition. These findings are highly consistent with conclusions from previous studies regarding the impact of demographic factors on PD cognitive function (Adhikari et al., 2012), further confirming the multifactorial pathogenesis of PD-related CI. Based on our results, we suggest that clinical practice should comprehensively consider key factors such as patient gender, age, disease duration, and education level to formulate individualized cognitive assessment plans and precise intervention strategies.

During serum protein component analysis, quantitative detection showed that serum INPP5D and FLNA expression levels were significantly lower in the PD group than in the HC group and were negatively correlated with the degree of CI. From a molecular mechanism perspective, INPP5D, as a key negative regulator of microglial activation, its downregulation may release the inhibition on neuroinflammation (Chou et al., 2023); while FLNA, as a cytoskeletal regulatory protein, its reduced expression may impair synaptic plasticity and neuronal migration ability (Binder et al., 2024). The coordinated downregulation of these two proteins may collectively weaken neuroprotective mechanisms, accelerating neuronal degeneration. Simultaneously, we observed significantly upregulated serum detection of ICAM-1, PCSK9, and JAK1 in PD patients, which positively correlated with the degree of CI. Mechanistic studies indicate that ICAM-1 can be significantly induced under the stimulation of pro-inflammatory factors such as TNF-α and IL-6 (Abruscato et al., 2025), and its elevation may reflect a systemic inflammatory state; PCSK9, besides affecting neuronal cholesterol homeostasis via the low-density lipoprotein (LDL) receptor pathway (Lagace, 2014), can also directly activate inflammatory pathways such as NF-κB (Tang et al., 2024); furthermore, JAK1, as a core component of the JAK–STAT pathway, its upregulation suggests the persistent activation of the neuroinflammatory response (Hong et al., 2024). Combining these findings, it is suggested that these three substances play a synergistic role in triggering PD-related inflammation. Notably, although LCAT expression showed no significant difference between PD and HC serum groups, subgroup analysis of PD-CI revealed a positive correlation with the degree of CI. This phenomenon may reflect that in the early stages of the disease, LCAT levels are regulated by liver function and basic metabolic status, but as the disease progresses, aggravated lipid metabolism disorders may lead to dynamic changes in LCAT expression. Based on these discoveries, we hypothesize that PD-CI may follow a cascade pathological mechanism of “neuroinflammation triggering—lipid metabolism dysregulation—cell adhesion disruption.” Future research should focus on elucidating its specific mechanisms of action across multiple levels, including system, organ, and cell.

Diagnostic efficacy analysis indicated significant limitations in the application value of single serum biomarkers for the differential diagnosis of PD. Evaluation via ROC curves revealed that INPP5D and LCAT exhibited high specificity but significantly insufficient sensitivity, suggesting they may be more suitable as auxiliary exclusion diagnostic indicators; FLNA and JAK1 showed relatively high sensitivity but low specificity, potentially leading to a high false-positive rate in clinical application; the areas under the curve (AUC) for ICAM-1 and PCSK9 approached random chance level and were not statistically significant, thus unsuitable as independent biomarkers. These results clearly demonstrate that relying on a single biomarker is insufficient to meet accuracy requirements for clinical diagnosis of PD presence. Future research should consider establishing multi-biomarker combined diagnostic models to optimize diagnostic performance by combining biomarkers with different characteristics. The exceptional AUC values for PCSK9 and LCAT in identifying PD-CI, while promising, must be interpreted with caution due to the relatively small sample size and lack of an independent validation cohort, which raises the possibility of model overfitting.

Further investigation focusing on cognitive dysfunction in PD patients found that the levels of the aforementioned serum proteins were closely related to the degree of CI. INPP5D and FLNA expression levels showed significant positive correlations with MoCA, MMSE, and CDR scores, suggesting their potential as positive indicators for cognitive function improvement; whereas ICAM-1, PCSK9, JAK1, and LCAT showed significant negative correlations, indicating they may exacerbate cognitive decline through inflammatory or lipid metabolism dysregulation pathways. This further confirms the important role of serum proteins in the pathological process of PD cognitive dysfunction. Notably, the association between MoCA scores and biomarkers was significantly stronger than with MMSE and CDR, demonstrating its sensitivity advantage in PD cognitive assessment. Concurrently, ROC curve analysis revealed the differential value of these six serum protein biomarkers in predicting PD-CI. PCSK9 and LCAT, combining high specificity and sensitivity, are the best candidate biomarkers for predicting PD-CI; FLNA is suitable as an auxiliary screening tool; although INPP5D and JAK1 were statistically significant, their low sensitivity necessitates combination with other biomarkers; ICAM-1 showed insignificant predictive value and weak association with PD-CI. Therefore, a dual-biomarker system of PCSK9 and LCAT could serve as an early diagnostic tool for PD-CI, particularly suitable for monitoring high-risk patients. Future studies could employ machine learning to integrate multiple biomarkers or clinical indicators to further enhance predictive accuracy.

Based on integrated pathway analysis, this study systematically elucidated the key molecular network characteristics in PD pathogenesis. KEGG pathway enrichment analysis revealed significant synergistic effects among signaling pathways such as “Focal adhesion,” “Leukocyte transendothelial migration,” and JAK–STAT in PD pathogenesis. Specifically, the widespread activation of the Focal adhesion pathway suggests disordered cell-matrix interactions, potentially leading to abnormal neurotrophic factor signaling and decreased neuronal survival; the abnormal activation of the Leukocyte transepithelial migration pathway reflects blood–brain barrier dysfunction, allowing peripheral immune cells to infiltrate the central nervous system and exacerbate neuroinflammation; although the JAK–STAT signaling pathway had a moderate Gene-Ratio (0.24), as a core regulatory hub, its persistent activation may drive chronic neuroinflammation and neuronal apoptosis. Further network topology analysis using Cytoscape identified ICAM-1 and ITGB2 as the central hub nodes of the entire interaction network. Molecular mechanism studies show that ICAM-1 not only participates in inflammatory responses by mediating leukocyte-endothelial cell adhesion (Bui et al., 2020) but can also promote the release of pro-inflammatory factors like TNF-*α* and IL-6 by activating microglia (Yun et al., 2025), forming a positive feedback loop for neuroinflammation. ITGB2 (integrin β2) synergistically regulates the leukocyte migration process with ICAM-1 (Li et al., 2025) and simultaneously affects microthrombus formation and cerebral microcirculation disorders through the “Platelet activation” pathway (Chen et al., 2025), potentially exacerbating ischemic damage to dopaminergic neurons in the substantia nigra. Notably, these two core nodes interact closely with key effector molecules such as STAT3 and JAK1, integrating pathological processes like neuroinflammation, cell adhesion, and immune cell infiltration into a unified molecular network. Based on these findings, this study proposes the following targeted therapeutic strategies: dual-target inhibition of ICAM-1/ITGB2 could simultaneously block leukocyte infiltration and microcirculation disorders; regulation of the JAK–STAT pathway may interrupt the vicious cycle of chronic neuroinflammation; a multi-target synergistic intervention strategy holds promise for achieving more comprehensive neuroprotective effects. These research outcomes not only deepen the understanding of the multi-system pathogenesis of PD but also provide an important theoretical basis and potential targets for developing precision treatment strategies based on molecular network regulation.

This study systematically compared differences in serum markers between PD patients and the HC group. The results showed no significant differences in demographic characteristics or basic medical history between the two groups, but PD patients exhibited significantly impaired cognitive function and mental health, confirming the presence of typical cognitive dysfunction and emotional disorders in PD. Abnormal expression of six serum proteins, correlated with cognitive function, provides important clues for understanding the molecular mechanisms of PD and developing novel diagnostic methods. While the diagnostic efficacy of a single serum marker for PD was limited, the six serum markers collectively showed good predictive efficacy for identifying CI in PD patients, indicating that integrating multi-omics data with clinical features holds promise for achieving early and precise diagnosis and individualized treatment for PD. In particular, PCSK9 and LCAT, as highly efficient predictive markers for PD cognitive dysfunction, possess significant clinical translational value. Furthermore, the study revealed that these proteins may play important roles in pathological processes such as neuroinflammation, cell survival, and signaling in PD. Future validation of these findings through larger, multi-center studies and exploration of their potential clinical application prospects are warranted.

Despite achieving important findings, this study has several limitations. First, as a cross-sectional design, causal relationships between serum protein changes and disease progression cannot be established. Second, the relatively small sample size may limit generalizability, and the lack of an external validation cohort further restricts the universality of the conclusions. Third, although severe psychiatric disorders were controlled for, mild to moderate anxiety and depression could still affect cognitive assessments; this should be considered when interpreting the results and highlights the need for future studies to explore the independent contributions of depressive and apathy symptoms to cognitive function in PD (Giannouli and Tsolaki, 2021). Additionally, potential confounding from genetic variants (e.g., APOE, PCSK9, LCAT) or concurrent lipid profiles, which may influence both serum protein levels and cognition, was not accounted for. Finally, the significant age difference between PD and healthy control groups, while common in such studies, represents a potential confounder. Future research should consider larger, longitudinal cohorts and integrative machine learning models combining biomarkers and imaging features to further elucidate PD pathogenesis and improve diagnostic strategies. Several additional limitations should be considered. All PD patients were receiving stable anti-Parkinsonian medication regimens during the study period. Although cognitive assessments were performed during clinically stable phases to minimize fluctuation-related variability, we cannot completely exclude the possibility that dopaminergic or other medications may have influenced serum biomarker levels or cognitive performance. Second, biomarkers such as PCSK9 and LCAT are known to be modulated by lipid metabolism, dietary intake, and lipid-lowering therapies (Pott et al., 2024; Yang et al., 2022). Future studies should incorporate comprehensive metabolic profiling and medication records to better delineate the specific contributions of these biomarkers to PD-related cognitive impairment.”

## Data Availability

The datasets presented in this study can be found in online repositories. The names of the repository/repositories and accession number(s) can be found in the article/Supplementary material.

## Footnotes

1
https://davidbioinformatics.nih.gov/

2
https://cn.string-db.org/
