# Supplementary material for: High-efficacy serum biomarkers PCSK9 and LCAT predict cognitive impairment in Parkinson’s disease
Source: Front Psychol. 2026 Apr 15;17:1789929. doi: 10.3389/fpsyg.2026.1789929 (PMC13124563; doi:10.3389/fpsyg.2026.1789929)
Supplement: Supplementary file 1 [file Table_1.docx]

**Table.S1** Binary logic system base on MoCA

|  | B | S.E, | Wals | df | Sig. | Exp(B) | 95% CI for Exp (B) | |
| --- | --- | --- | --- | --- | --- | --- | --- | --- |
|  |  |  |  |  |  |  | Lower | Upper |
| INPP5D | 0.008 | 0.009 | 0.778 | 1 | 0.378 | 1.008 | 0.990 | 1.026 |
| FLNA | 0.046 | 0.026 | 3.277 | 1 | 0.070 | 1.047 | 0.996 | 1.101 |
| ICAM1 | 0.031 | 0.016 | 3.431 | 1 | 0.064 | 1.031 | 0.998 | 1.065 |
| PCSK9 | -0.075 | 0.053 | 1.969 | 1 | 0.161 | 0.928 | 0.835 | 1.030 |
| Age | -0.046 | 0.053 | 0.752 | 1 | 0.386 | 0.955 | 0.860 | 1.060 |
| LCAT | -0.276 | 0.128 | 4.620 | 1 | 0.032 | 0.759 | 0.590 | 0.976 |
| HAMD | 0.120 | 0.192 | 0.389 | 1 | 0.533 | 1.127 | 0.774 | 1.643 |
| Education | 0.227 | 0.371 | 0.374 | 1 | 0.541 | 1.255 | 0.606 | 2.596 |
| Gender | -0.277 | 1.117 | 0.061 | 1 | 0.804 | 0.758 | 0.085 | 6.773 |
| JAK1 | -0.159 | 0.102 | 2.452 | 1 | 0.117 | 0.853 | 0.699 | 1.041 |
| Disease duration | 0.097 | 0.129 | 0.561 | 1 | 0.454 | 1.102 | 0.855 | 1.420 |
| HAMA | -0.226 | 0.197 | 1.316 | 1 | 0.251 | 0.798 | 0.543 | 1.173 |


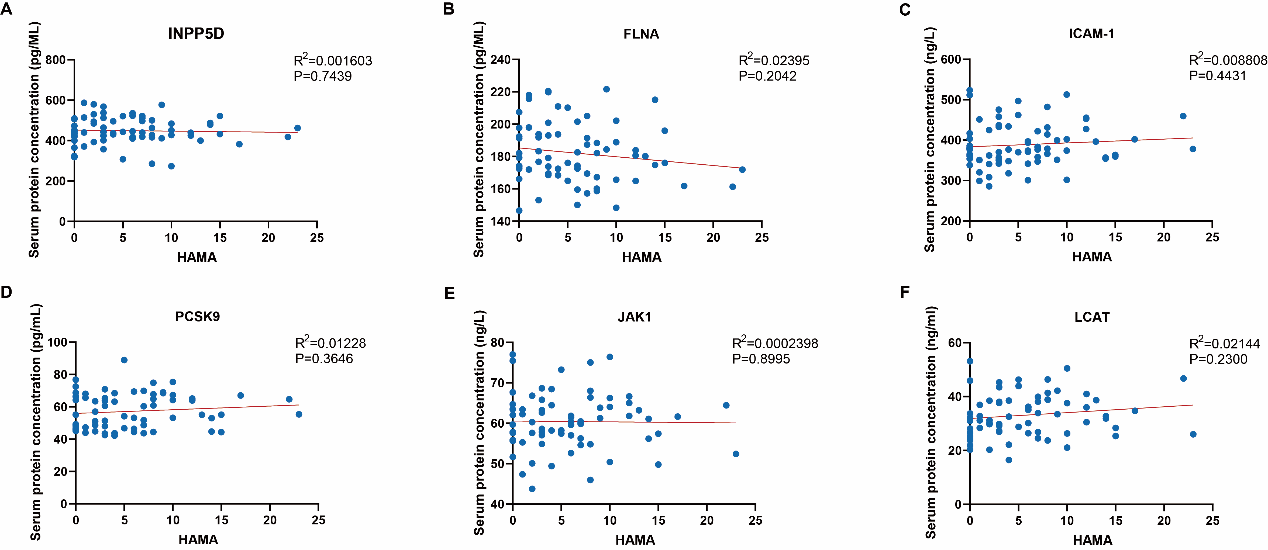


**Fig.S1** The correlations between serum protein levels and HAMA scores in Parkinson’s disease patients (n_PD_=75). (A) INPP5D, (R^2^=0.001603, P=0.7439); (B) FLNA, (R^2^=0.02395, P=0.2042); (C) ICAM-1, (R^2^=0.008808, P=0.4431); (D) PCSK9, (R^2^=0.01228, P=0.3646); (E) JAK1, (R^2^=0.0002398, P=0.8995); (F) LCAT, (R^2^=0.02144, P=0.2300).


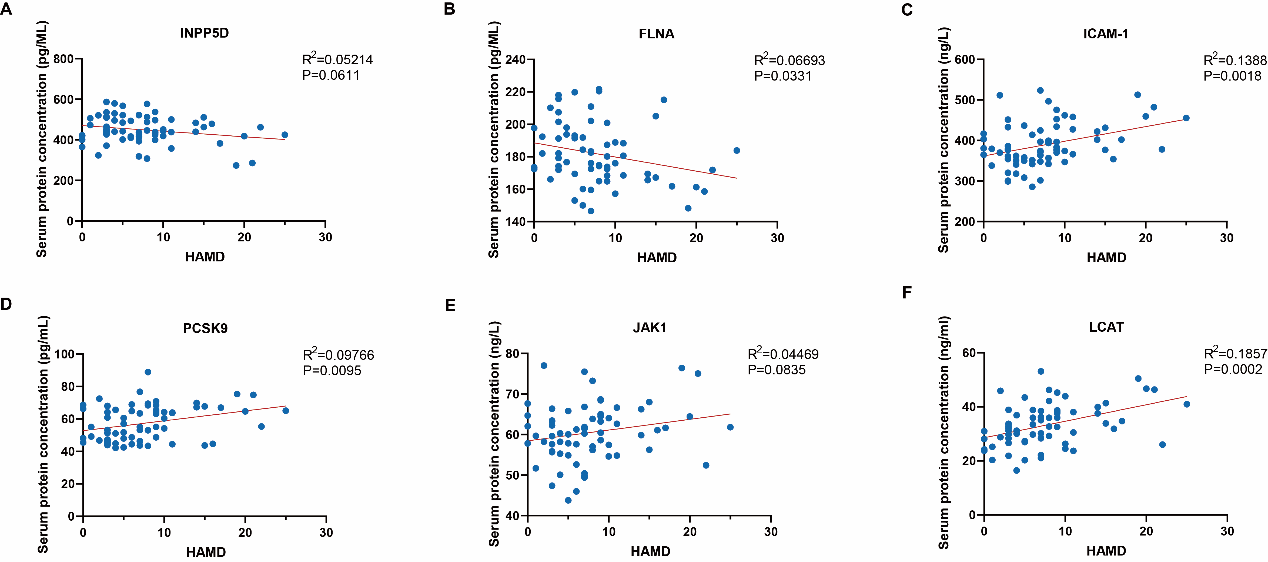


**Fig.S2** The correlations between serum protein levels and HAMD scores in Parkinson’s disease patients (n_PD_=75). (A) INPP5D, (R^2^=0.05214, P=0.0611); (B) FLNA, (R^2^=0.06693, P=0.0331); (C) ICAM-1, (R^2^=0.1388, P=0.0018); (D) PCSK9, (R^2^=0.09776, P=0.0095); (E) JAK1, (R^2^=0.04469, P=0.0835); (F) LCAT, (R^2^=0.1857, P=0.0002).
